# The Role of Emotional Granularity in Critical Reflexivity: A Reflexive Diary Study

**DOI:** 10.3390/bs16020279

**Published:** 2026-02-14

**Authors:** Valentino Zurloni, Giulia Tossici, Raffaele De Luca Picione

**Affiliations:** 1 “Riccardo Massa” Department of Human Sciences for Education, University of Milano-Bicocca, 20126 Milan, Italy; giulia.tossici@unimib.it; 2 Faculty of Law, Giustino Fortunato University, 82100 Benevento, Italy; r.delucapicione@unifortunato.eu

**Keywords:** emotional granularity, critical reflexivity, emotional competence, cognitive processes

## Abstract

The paper aims to explore the relationship between emotions and reflexivity, with reference to the constructs of critical reflexivity and emotional granularity. These two constructs and their operationalization constitute the theoretical–methodological background of an empirical exploratory research study conducted on a sample of adult workers aged between 18 and 55, who were subjected to a diarist-style reflective writing course. The overall aim of the course was to ascertain whether, how and to what extent reflective practices of the narrative type can influence and modulate the stress response, both from the point of view of the participants’ assumption of awareness and from the point of view of the adoption of new behaviors. The central question that the present article proposes to discuss is related to the exploration of what the basic requirements/skills are on which the development of critical reflexivity is built over time, with particular attention to the role played by emotional competencies. This aspect represents one of the most relevant gaps in current research on critical reflexivity, which is severely limited by a general tendency towards the hyper-cognization of the models of analysis adopted in much of the research devoted to reflexivity, as well as by the little space given to the investigation of the emotional dynamics at play in its onset processes. The study carried out represents an initial exploration of this aspect, testing two main hypotheses: (a) the possibility of identifying and describing a preliminary threshold to the manifest development of critical reflexivity, prior to the development of process reflexivity; (b) the possibility that crossing this threshold may be facilitated by the acquisition of a good level of emotional competence, measurable through the emotional granularity construct. In the light of the quali-quantitative analyses carried out on the diaristic corpus, the hypotheses put forward have all been confirmed, consolidating the line of research aimed at investigating the role played by emotional competence in the development of critical reflexivity, in interaction and combination with the increasingly complex structuring of the cognitive processes underlying reflexivity.

## 1. Introduction

Research on reflexivity, and mainly on critical reflexivity, has found wide recognition and resonance within the scientific community from the 1980s and 1990s to the present. The topic cuts across psychology and the educational sciences in a variety of declinations and areas, including general psychology, social and work/organizational psychology, adult pedagogy, experimental pedagogy and didactics. If, however, one delves deeper into the problem of the genesis of this capacity and its developmental levers, it soon emerges how the theme is potentially more extensive and can benefit from a view capable of reading the evolutionary dynamics at play, through comparison with developmental psychology and dynamic psychology, as well as with the knowledge acquired thanks to recent developments in neuroscientific research on the subject of learning, cognition and affectivity.

Among the various theoretical traditions of reference concerning the topic of critical reflexivity (Lundgren & Poell, 2016; Taylor, 2017; Van Woerkom, 2010), the psychotherapeutic tradition occupies a particularly prominent place in the thought of Mezirow and his theory of transformative learning (Mezirow, 1990, 1991), which represents the main theoretical reference for the analyses conducted in this paper. In his work, Mezirow defines critical reflexivity as “reassessing the way we have posed problems and reassessing our own orientation to perceiving, knowing, believing, feeling, and acting” (Mezirow, 1990; Van Woerkom, 2010). To this theoretical lineage, one must add the influence exerted on Mezirow by theories of organizational learning (Argyris & Schön, 1996) and the equally relevant theoretical debt to pragmatic constructivism, particularly Dewey’s (1933) pioneering reflections on reflexivity and its role in adult education.

But as much as the psychotherapeutic tradition is more predisposed to recognize and study the role of unconscious and affective dynamics, it too is influenced by the same rationalistic and idealistic prejudice that tends to characterize critical reflection as a systematic cognitive process, inspired by the laws of traditional and deductive logic and conceptualized as an essentially rational means, useful for the re-evaluation of problems and our perception of them (Van Woerkom, 2010). This has hindered the adequate appreciation of the affective and emotional components that accompany and support the development of reflexivity, disregarding their role as powerful activators of the processes of personal transformation and change that allow critical reflexivity to fully unfold (Van Woerkom, 2010; Taylor, 2017).

One of the major problems in conceptualizations of critical reflexivity is the almost exclusive focus on conscious thought and knowledge understood in a declarative, verbal and explicit sense (Van Woerkom, 2010):

“*When we compare the definitions and ideals of critical reflection, we can observe a common bias of instrumental rationality in the definitions of critical reflection. All definitions implicitly characterize critical reflection as a systematic cognitive process that is targeted toward a specific ideal (…) It is assumed that a person, or a rational agent, has a system of beliefs and a set of goals or desires or intentions. The norms of rationality require that agents only make decisions in accordance with their beliefs and their current perceptual inputs and combine rules and beliefs according to the principles of deductive logic. As all traditions use critical reflection as a means to achieve specific ideals, critical reflection can be seen as a form of instrumental rationality.*”

(p. 343)

Taylor (2017) argues that this common assumption in favor of instrumental rationality as the foundation of critical reflexivity has hindered the appreciation of the contribution of emotions and affections in research on critical reflexivity. Several studies have shown that critical reflection and rationality are often granted too much importance in perspective transformation and that intuition, other ways of knowing, emotions, and empathy are of equal importance (Taylor, 1997). In support of this observation, Swan and Bailey (2004) also point out that the relationship between emotion and reflection has until recently been largely underanalyzed and undertheorized in the literature on reflection. In their research, conducted through interviews with a population of managers, the two scholars discovered that emotions are often catalysts for reflection. This is in line with the review of empirical studies conducted by Taylor (1997), which reveals that transformative learning is not just rationally driven but also relies strongly on the exploration and resolution of feelings.

The bias that affects emotions, intuitions, and affections in the field of reflection is accompanied by an almost exclusive focus on conscious thought in conceptualizations of critical reflection (Van Woerkom, 2010). However, there are unconscious aspects of experience that play a decisive role in learning (Tossici & De Luca Picione, 2024; Tossici et al., 2024), as highlighted by affective neuroscience, which is consistent with the thinking and models developed by psychological research of an evolutionary matrix and psychodynamic orientation (Crittenden, 1994, 1999; Crittenden & Landini, 2008; Fonagy, 2018; Fonagy & Target, 1997). Furthermore, empirical studies have shown that this type of learning comes into play in the acquisition of complex models and that implicit knowledge exceeds what can be verbalized (Jiang & Chun, 2001; A. S. Reber, 1993; P. J. Reber et al., 2003). Taylor (1997) also noted that, in many cases, meaning structures can be altered at a non-conscious level outside the individual’s awareness, without rational and deliberate examination of hypotheses. Further field research conducted by Ball (1999) confirmed this idea: many of the study participants reported that their transformative experiences occurred beyond any explicit exercise of reflection and, therefore, happened “inconspicuously, perhaps even unconsciously, and in the context of everyday activities” (Ball, 1999, p. 261). One interpretation of this process can be found in the idea of Burgoyne and Hodgson (1983), according to whom transformation mediated by reflection occurs gradually, through a process of tacit learning that replaces one belief with another through a progressive accumulation of evidence and experiences (Van Woerkom, 2010).

Implicit learning, therefore, still remains a largely unexplored field of research, covering the vast domain of knowledge that is acquired on this side of conscious attempts to learn and, to a large extent, in the absence of explicit knowledge of what has been acquired (Eraut, 2000; A. S. Reber, 1993). Being implicit, however, it is not readily available to consciousness and tends not to be available for critical reflection. This probably explains the difficulty on the part of different research traditions in focusing on its presence and appreciating its importance within the explicit manifestations—in terms of thought, language, and actions—of reflexivity, including, of course, critical reflexivity.

The present article intends to contribute to bridging this gap by presenting a series of theoretical reflections supported by empirical data that were collected within the exploratory research trial “Getting to know yourself better to be better. Reflective and psycho-corporeal practices for personal well-being”, carried out by a group of researchers from the University of Milan Bicocca (CE Prot. No. 0180050 of 02/05/2024-UOR: 003406). This trial involved a sample of workers who were proposed a diaristic pathway aimed at investigating their levels of reflexivity through a coding grid inspired by Mezirow’s (1990, 1991) construct of critical reflexivity, combined with that of emotional granularity proposed by Barrett (2017), for the exploration of the underlying affective and emotional components.

In the following paragraphs, the theoretical and methodological framework of the research will first be outlined, and then the results obtained will be shared and discussed.

## 2. Theoretical Foundations

In the elaboration of his theory of transformative learning, an important turning point for Mezirow was the rediscovery of Dewey’s work and his definition of reflection as active, persistent, and careful consideration of any belief or presumed form of knowledge in the light of the reasons which support it and the further conclusions to which it tends (Dewey, 1933). It is on this basis that Mezirow draws the capital distinction between *unreflective* and *reflective action*. The former includes all human actions that are *habitual* (i.e., linked to automatisms) or *thoughtful* (which also includes *introspection*) without requiring the intervention of reflection in order to take place; the latter, on the other hand, includes actions in which the subject comes to evaluate the foundations of his or her own beliefs (Mezirow, 1990) and verify their validity (Kember et al., 2000), thus making a critical examination of the *content*, *process* or *premises* of our efforts to interpret and give meaning to an experience (Mezirow, 1991). Such ‘criticism’ can thus invest thought and things in different ways and at different levels:

1. *reflection on the content*: when we carry out a critical examination of the content or description of a given problem, remaining focused on the objective data of the same; it is a reflection on what we perceive, think, feel and act (Taylor, 2017) that answers the question “*What* is going on here? What is the problem?” (Cranton, 2006);
2. *process reflection*, which focuses on problem-solving strategies and thus on subjective choices of approach and method, rather than on its objective content per se. It is a reflection on how we perform the functions of perception that asks questions such as “*How* did this come about?” (Cranton, 2006);
3. *reflection on premises*, which takes place when the underlying assumptions or the problem itself are questioned, thus affecting the models we use to think, make decisions, implement actions, evaluate and weigh things. It is a reflection that implies becoming aware of *why* we perceive, think, feel or act as we do (Mezirow, 1991) and can be likened to Argyris and Schön’s (1974) concept of double-loop learning. Only it, according to Mezirow, corresponds to the notion of *critical reflexivity*.

This tripartition has been very successful among researchers. At the beginning of 2000, Kember proposed its operationalization by means of two different rating scales (Kember et al., 1999, 2000) and the creation of a questionnaire designed to measure levels of reflexivity in students, resulting in a model that is among the best known and most widely used in the field of psychology and educational sciences.

The choice to focus on this theoretical model and on Kember’s methodological approach stems from the conviction that Mezirow’s tripartite conception is particularly useful for achieving two objectives relevant to our research:

1. to bring out the aspects of tacit knowledge^1^ and the implicit learnings that are inherent in the explicit dimension of thought and knowledge;
2. to develop authentic reflection on premises.

### 2.1. Models and Methods of Explicating the Implicit Learnings of Reflective Knowledge

The first objective is related to the need to achieve a deeper and more satisfactory understanding of the nature of the *premises* that form the core of transformative learning theory. By *reflection on premises*, Mezirow means not a simple revision of *content* or *thought processes* and the mere resolution of specific problems, but rather the occurrence of a *perspective transformation,* in which the subject accesses the ‘*whys*’ underlying his or her own mental schemata. As Kember himself points out,

“*To undergo a perspective transformation it is necessary to recognize that many of our actions are governed by a set of beliefs and values that have been almost unconsciously assimilated from the particular environment. Premise reflection then requires a critical review of presuppositions from conscious and unconscious prior learning and their consequences.*”

(Kember et al., 2000, p. 385)

The *what and how of* people’s reasoning, perceptions, conferring of meaning, problem-solving and problem-posing, as well as the type of relationships they have with their physical and social environment, is related to a setting of *mental models* that hermeneutically frame these processes (Mezirow, 1990). Mezirow calls them meaning *schemes* or *perspectives*:

“*Meaning schemes are sets of related and habitual expectations governing if-then, cause-effect, and category relationships as well as event sequences.*”

“*Meaning perspectives are made up of higher-order schemata, theories, propositions, beliefs, prototypes, goal orientations, and evaluations, and what linguists call “networks of arguments.*”

“*Meaning perspectives refer to the structure of assumptions within which new experience is assimilated and transformed by one’s past experience during the process of interpretation. They involve the application of habits of expectation to objects or events to form an interpretation.*”

(Mezirow, 1990, p. 2)

If we retrieve and integrate the psychodynamic and evolutionary lessons, as Mezirow (1990, p. 2) explicitly does, we discover that such assumptions are mostly unconscious and related to implicit learnings matured in early life stages that are realized, first and foremost, within affectively connoted primary relationships:

“*Meaning perspectives are, for the most part, uncritically acquired in childhood through the process of socialization, often in the context of an emotionally charged relationship with parents, teachers, or other mentors. The more intense the emotional context of learning and the more it is reinforced, the more deeply embedded and intractable to change are the habits of expectation that constitute our meaning perspectives*”

(Mezirow, 1990, pp. 3–4)

These are ‘premises’ that constitute a sort of mental map, with a strong affective matrix, which the individual carries with him throughout his existence and which profoundly influences the subsequent cognitive processing carried out by the subject^2^, even the most complex and sophisticated ones, apparently entirely conscious and rational (Tossici & De Luca Picione, 2024; Tossici et al., 2024).

Contrary to the distrust that rationalistic prejudice has towards emotional processing, scientific and psychological research, in fact, has amply demonstrated how emotions are powerful ‘influencers’ of the organism’s cognitive evaluation and psychobiological regulation processes.

On the one hand, as LeDoux (2002) points out, they are one of the transversal organizers of psychic life, capable of orienting, guiding, and directing even subsequent cognitive appraisals and the main processes involved in attention, perception, memory and decision-making.

On the other hand, they significantly affect synaptic plasticity processes, i.e., the gene transcription mechanisms by which new memories are formed or pre-existing memories are reinforced in processes of *long-term potentiation*^3^ (Kandel et al., 2000; Nicoll, 2017). This implies that the processes of appraisal and *arousal*, i.e., the fundamental components of emotion, are closely and inextricably intertwined with the representational processes of ‘thinking’ and learning, so that no artificial or didactic boundaries can be created between thinking and emotion (Siegel, 2021).

The aspect that makes them almost invisible to self-reflection, however, is due to the fact that such unconscious mental schemata are so ingrained in people’s minds and bodies that they are very difficult to verbalize and analyze, giving rise rather to automatisms that tend to define the belief and value systems that orient the subject’s thinking, modes of action and relationships, as psychotherapeutic experience has highlighted at the clinical level, and psychological and neuroscientific research has justified on a theoretical and scientific level^4^ (Caruana & Viola, 2018; Salvatore et al., 2022; LeDoux, 2002; Kandel, 2010). Bringing them out, therefore, is not easy and requires deep self-knowledge. To facilitate their explication and integration on a conscious level, the key aspect to work on is the emotions and affective components that support and nourish them and that constitute the amalgam from which these patterns take shape.

The work on emotions and the affective aspect of subjective experience thus proves to be decisive for the development of a critical reflexivity that seeks to recognize and move flexibly between the assumptions and premises linked to one’s own implicit reference models. This focus should go hand in hand with the attention so far paid to the rational and conscious components of reflexivity.

Given that the transformation of the models through which we think, act, make decisions, and evaluate the attitude to take towards things, people and situations, goes a long way towards the development of good emotional competence, we considered it essential to supplement Mezirow’s model of critical reflexivity with a construct capable of assessing the quality of this emotional competence. The choice fell on the concept of emotional granularity proposed by Lisa Feldman Barrett. Barrett (2017) defines emotion granularity as an individual’s ability to create different and context-specific categories of emotions, and thus to perceive and differentiate different nuances of emotions. The term ‘emotional granularity’^5^ is a coinage that Barrett picked up in the 1990s from the domains of computer science and physics, domains in which it denotes a certain level of detail, e.g., coarse-grained or fine-grained, of the available data sets or phenomena studied. This coinage, applied to psychology, has inspired a new research domain in the field of emotion.

In Barrett and Russell’s (2014) model, emotion is defined by the intertwining of hedonic valence (positive/negative) and intensity of emotional experience, and granularity indicates the ability to express feelings in words with a high degree of specificity and precision (Barrett, 2017).

The dimensions that nurture and structure this capacity are represented by

the *emotional lexicon*, i.e., the number of emotional concepts available to express one’s internal states;
the level of *differentiation* of emotions, i.e., the ability to grasp the nuances of the same emotional category;
*emotional specificity*, i.e., the ability to describe emotion in a specific, precise and concrete manner.

These three dimensions, together with the subject’s ability to use emotions in a similarly differentiated and specific manner to enhance his or her emotional regulation, underlie the idea that greater emotional granularity is a protective factor for mental and physical well-being, as it makes emotional responses more specific and differentiated, increases people’s level of awareness of their emotional life, and facilitates learning and change pathways mediated by more effective emotion regulation.

The confirmations gathered over time in favor of this hypothesis (Hoemann et al., 2020; Smidt & Suvak, 2015) convinced us to choose this construct and operationalize it in our empirical analysis, first by conducting a parallel and then cross-validation of the levels of reflexivity and emotional competence of the diary entries examined. The aim was to test the validity of the hypothesis that diaristic entries showing more evolved levels of reflexivity were also an expression of a more mature and complex level of emotional competence and mastery; the results of which are reported in the second part of this paper.

### 2.2. Activating Factors for the Perspective Transformation of Critical Reflexivity

It has already been emphasized that the pathway of explicating *the tacit knowledge* inherent in reflexive processes represents a long and complex process in which the cognitive and affective components, as well as the conscious and unconscious aspects of subjective experience, must walk together. But how is this path of self-awareness constructed, and what activates the perspective transformation at play in critical reflexivity?

The genesis of this concept may offer some useful indications to identify where the turning point between non-reflexivity and critical reflexivity lies. It is useful to keep in mind the distinction outlined by Mezirow around the late 1980s—i.e., between the first and second formulations of his transformative learning theory (Taylor, 2017)—between two different ways of looking at things and problems. On the one hand, *objective reframing* (CRA), in which reflection on assumptions is based on universal standards of rationality and discourse, occurs when critical reflection takes the form of a critical examination of the assumptions on which something is based. On the other hand, *subjective reframing* (CSRA) indicates reflection on what caused the very occurrence of the assumptions on which those assumptions are based, requiring analysis of the psychological or cultural assumptions of our belief and value systems (Taylor, 2017).

If, over time, the distinction between these two modes of critical reflection fades to the point of collapsing CSRA entirely into CR (critical reflection) (Taylor, 2017), this distinction, while reabsorbed within the logical–methodological framework of Mezirow’s theory, remains a fundamental implicit juncture of his theoretical system because it marks the transition of reflection from an *objective* focus—looking at the things and the *what, the quid*, about which one reflects—to a *subjective* focus, looking at the subject and the *way in which* evaluative processes take place (the *how*). Without this transition, the path to critical reflection would inevitably remain blocked.

However, this transition, on closer inspection, takes place *before* its integral completion in critical reflection. Its first appearance takes place when the subject comes to integrate the critical examination of the *content* of experience with that of the *process* and the specific modalities of its elaboration. It is at that moment, in fact, that an observational shift occurs for the first time from the *what* (reflection on the content) to the *how* (reflection on the process) of the experience that opens up to the subject the exploration of the vast field of his or her inner resources of signification, in terms of thought and of the ways of processing and elaborating the experience. These mental gymnastics, if exercised over time, give the subject the ability to differentiate and move gradually with greater flexibility between the objective scope of the factual aspects of the world and the entirely subjective and interior capacity to rework those ‘facts’ and construct new realities. This power characterizes the human as a *symbolic animal* (Cassirer, 1953, 2023) that sweeps with the infinitude of the cultural resources of its mind into the domain of the *possible*, going beyond the limits and constraints of the pure reality and *factuality* of the objective world.

Our idea is that it is precisely in the discovery of this ‘option’ that the transformative power of Mezirow’s critical reflection takes root and takes shape. In fact, it is only after gaining access to the vast field of symbolic formation that the subject discovers he can remodulate, often in a very incisive manner, the terms in which reality is thought, experienced, conceptualized, and understood; and thus that he can modify that very *factuality* that previously appeared as *external* to the subject and, in some way, unbreakable and unalterable. Proceeding in this direction and refining his own cultural sensibility over time and with practice, the subject comes to understand that he has within himself the power to reform reality according to his own modes of elaboration.

## 3. Methodological Foundations

### 3.1. Reflective Diary

Various techniques are described in the different models of reflection, such as the use of diaries, questioning skills and group work to facilitate learning at different learning stages. Boud et al. (1985) suggest using diaries and journals in the early stages of learning. Stockhausen (1994) proposes using diaries at all stages of the reflective learning cycle to record thoughts and feelings. Johns recommends keeping a structured diary to allow for periodic reviews of the experience and to make sense of learning over time. Each model provides less detail on how reflective learning in the later stages of the cycle can be facilitated. It appears that group work (Stockhausen, 1994) and discussion and questioning (Boud et al., 1985; Johns, 1994; Stockhausen, 1994) take on a more important role as learners progress through the stages of learning. However, even less research is available on these methods (Platzer et al., 1997).

The habit of keeping and writing a diary to improve self-awareness and cope with difficult times has been well documented in several works of literary fiction. Keeping a diary creates a narrative of events, thoughts, hopes and emotions on an intensely private level. It can be seen as an extremely pure form of self-reflection, not intended to be shared (Travers, 2011). A key aim of this study is to explore the uses of reflective diary writing by diarists who are asked to recount problematic events.

The scholarly tradition in the field of psychology that explores the use of diaries to increase well-being and achieve physical and mental health benefits can be traced back to the work of Pennebaker (1993, 1997, 2004) and colleagues (e.g., Pennebaker et al., 1987, 1988, 1990; Pennebaker & Segal, 1999). These studies indicate that getting people to write about their emotions and deep thoughts, particularly about traumatic and stressful events, can lead to improvements in social, psychological, behavioral and biological areas (Kacewicz et al., 2007). Pennebaker and Segal (1999) suggested that uncovering the self through writing can promote the construction of narrative pathways of meaning and that writing is a way to make sense of one’s life experience and bring together otherwise fragmented stories, memories and experiences.

Rather than focusing on the expressive aspects of writing, more recent studies have used the diaristic technique to promote forms of learning and styles of reflexivity, shifting the emphasis to the process rather than the product (Rolfe et al., 2001; Loo & Thorpe, 2002). To facilitate the development of processes related to reflectivity and reflective learning, many professional training programs involve learners in writing reflective diaries as one of their learning activities (Conner-Greene, 2000; Patton et al., 1997; Woodward, 1998). Past research outlines that writing reflective diaries improves reflection, critical thinking, the integration of theory with practice, and promotes professional growth (Brown & Sorrell, 1993; Callister, 1993; Kea & Bacon, 1999; O’Rourke, 1998). As an assessment method, reflective diaries not only provide evidence of understanding of content knowledge, reflection, professional judgment and application, but also enhance critical self-reflection and self-awareness (Biggs, 1999; O’Rourke, 1998) and improve learner assessment performance (Conner-Greene, 2000).

In terms of skills development, writing can increase awareness of the importance of choices at work, the symbolic and metaphorical meaning of words, what is important to pay attention to, and how to personalize word choice in public communication contexts. A diary helps to support oneself emotionally at work and can help diarists reflect on experiences, thus providing a way to address the gap between theory and practice (Fonteyn & Cahill, 2001; Hancock, 1999). The diary can also help develop narrative skills, see different possibilities for choice and action in a situation, and increase observational skills (Callister, 1993). By writing, thoughts transferred onto paper help analyze events in a less personal and more objective way. Furthermore, the process of mentally constructing words and sentences before they are fixed on paper allows events to be reconstructed in a more structured and accurate manner (Travers, 2011).

The literature has shown that reflective diaries are useful tools for facilitating reflective thinking and reflective learning (Tang, 2002). Writing a diary requires the diarist to think back on events that have taken place and provides an opportunity to express personal thoughts. When used in a learning context, reflective journals offer users the opportunity not only to rethink learning activities, explicitly and purposefully identifying what they have learned, but also to relate what they have learned to their practice, evaluate their practice in light of theories, and formulate action plans for improvement. The very nature of a diary also allows users to explore and express their learning in a personal way, making that learning take on personal meaning and be useful in their own context. Reflective diaries, either as a stand-alone activity or as part of a portfolio, could be implemented as a learning and assessment tool to facilitate and assess reflection effectively (Tang, 2002).

### 3.2. Reflective Diary Task

When conducting a diary study, there are several methodological aspects to consider. Among these, Nezlek (2012) highlights some fundamental ones:

- Type of diary, linked to the research question: In the case of intra-individual aspects that link a psychological state to external conditions (i.e., anxiety–stress), interval or signal-contingent protocols are recommended (the diary is written not when an event occurs but at pre-established intervals). The frequency is dictated by how often the target event/state is expected to occur. While it can provide detailed descriptions of an individual’s changes in relation to the target, it does not provide many details about each occurrence.
- Duration of treatment: The lower the frequency of occurrences of a target event/state, the longer the duration of treatment. The smaller the sample, the more advisable it is to extend the treatment (increasing the number of repeated measurements). As a rule, two weeks is considered an adequate duration. The more complex the protocols, the more resources the diarist must bring into play, and the more complicated it becomes to maintain compliance.
- Frequency of writing: The higher the frequency of writing, the lower the amount of data that can be acquired. It is essential to make a realistic estimate of the time the diarist should devote to each entry to determine the frequency. Regardless of the type of diary and the events to be observed, the less time there is between the event and the entry, the better (this reduces the risk of unwanted variables). It should be noted that writing the diary should not affect the organization of the diarist’s activities.
- Amount of information to be collected for each entry: The more aspects the diarist is required to cover, the less accurate and in-depth their reports will be on each aspect. The constructs behind each aspect should be clarified as much as possible to the diarist to increase compliance.

In the present study, to facilitate and structure the writing of the reflective diary, the diary form to be completed was accompanied by a handover form and a form containing some guidelines to guide the participants in their reflective writing.

*Handover form*: Each administration of the reflective diary was accompanied by a handover form summarizing the purpose, time and manner of the diary activity the participants were asked to carry out. The narrative diary was presented as an activity that allows one to document one’s daily actions, collecting experiential material useful for activating a process of listening and self-reflection in everyday life. In particular, the narrative diary is a very useful tool to

strengthen analysis and observation skills,
develop capacities for processing one’s own experience,
focus on contradictions in thought, and
give voice/expression to one’s emotions

The participants were asked to reflect on an event that happened to them at work or in their personal lives that generated tension and/or problems during the week.

To help the participants identify salient events, the main inclusion and exclusion criteria were indicated. Specifically, the participants were asked to talk about something that

is within the ordinary and not the extraordinary (exclusion of traumatic events);
happened, preferably, in the current week or in the week just before; in any case, at least 24 h after the moment of writing.

In the absence of such an event occurring in the current week or the week directly preceding it, the participants were asked to describe an event occurring in the past as recently as possible.

The participants were instructed that entries were free and unrestricted in length; however, to facilitate the development of the narrative, the diary was accompanied by some guidelines that acted as prompts and stimuli for reflection.

It was also emphasized that the confidentiality of the diary entries was guaranteed and that they would only be used for statistical and scientific purposes, in a completely anonymized form.

Finally, since the completion of the diaries took place online via Google Form, the participants were invited to contact a specially created help desk to handle any doubts about the instructions received or to resolve technical difficulties with the form.

Guidelines to guide participants in the reflective writing technique are given in Table 1.

The preliminary operation was to explore how narrative thinking is structured through the technique of reflective diaristic writing (Study 1). Secondly, the study set out to observe and analyze whether and how reflexive practices interact with the linguistic-symbolic articulation of emotional experience. Indeed, it was assumed that narrative processes can train the mind to help reflexively manage emotions (Study 2).

## 4. Study 1: Exploration of the Reflexive Structure of Thought in Diaristic Entries

### 4.1. The Coding Grid

To explore how narrative thinking is structured through the technique of reflective diaristic writing, we carried out a qualitative content analysis on the textual corpus produced by the participants. The coding grid proposed by Kember et al. (2000) was used to identify and assess levels of reflexivity, of which an adapted and partially modified version is proposed. In summary, Kember proposes the following 7 categories:

(1) *Habitual action* (HA). Habitual action consists of what has been learned previously and through frequent use becomes an activity that is performed automatically or with little conscious thought.
(2) *Introspection* (I). Introspection intercepts the affective domain. It refers to feelings or thoughts about ourselves or feelings about others. Introspection remains at the level of recognition or awareness of these feelings.
(3) *Thoughtful action* (TA). Thoughtful action makes use of existing knowledge without evaluation of that knowledge, so that learning remains within pre-existing patterns of meaning and perspectives. Thoughtful action can be described as a cognitive process that includes knowledge, understanding, application, analysis and synthesis.
(4) *Content reflection* (CR). Content reflection is about what: reflection on what we perceive, think, feel or act.
(5) *Process reflection* (PR). Process reflection is about how, i.e., how we think. It involves an analysis of how we perform the functions of perceiving, thinking, feeling or acting and an evaluation of the effectiveness in performing them.
(6) *Content and process reflection* (CPR). Mezirow’s original coding scheme was expanded to include a combined category for content and process reflection after Kember et al.’s (2000) evidence found examples of reflections in which content and process were inextricably linked.
(7) *Premise reflection* (PREM). Premise reflection is about a significant shift in perspective. It involves becoming aware of why we perceive, think, feel or act in a certain way.

Kember et al. (2000) claim that the level of reflective thinking increases from the bottom up. The first three coding categories (Habitual Action, Introspection, Reflective Action) denote non-reflective actions based on Mezirow’s (1991) work. Categories 4–7 (Reflection on Content, Reflection on Process, Reflection on Content and process and Reflection on Premise) represent levels of reflective thinking, where categories 4–6 are on the same level and category 7 is considered a higher level of reflection (Bell et al., 2011).

### 4.2. Sample and Diary Entries

Following the approval of the Ethics Committee of the University of Milano-Bicocca, we invited workers from two Italian companies, one public and one private, to participate in our study. In total, 18 workers, all Caucasian from North and Central Italy (14 males and 4 females; age range 20–60 years), joined the research voluntarily. The participants were asked to complete one diary entry per week for four consecutive weeks, guaranteeing anonymity on any published data obtained from the study. The resulting body of data comprises a total of 62 diary entries (not all participants produced the required 4 entries). Each entry was subjected to coding by two independent coders.

### 4.3. The Coding Process

Each diaristic entry was completed by the participants by filling in an online form, and then sent to the researchers at the end of each writing week. Each entry was subjected to a pseudonymization procedure to ensure the anonymity of the participants. To construct an ad hoc grid capable of adapting to the nature of diaristic writing, the researchers proceeded as follows:

Each coder became familiar with the coding grid of Kember et al. (1999) and applied it to a diaristic sub-sample (identical for each coder). Coding was performed line by line within the diary sub- sample. Subsequently, each coder assigned a reflexivity value to each diary entry. This approach was subsequently extended to the coding of all diaries to measure inter-rater reliability.

After coding the diaristic sub-sample (the total diaristic entries of a participant), the coder’s work was compared, discussed and resolved in cases of discordant coding. Subsequently, portions of text were selected and extrapolated to be added to the coding grid for illustrative and explanatory purposes.

This process led to the revision of Kember et al.’s (1999) coding grid as follows:

We excluded HAs because they were not relevant with respect to diaristic productions; the applicability of the dimension ‘habitual actions’ to textual productions has already originally been discussed by Kember et al. (1999) and in Bell et al. (2011). Additionally, no case of application of this dimension to the narrative production under consideration was found.

We noticed that some diaries presented multiple styles of reflexivity. In these cases, we determined to attribute to the diary the dimension of reflexivity that was predominantly used (based on the number of times it appears within the text and the extent of the text in which this strategy is used).

To better adapt the coding grid to the specificities of the narrative format of the reflexive diary, we reduced the grid to include 4 dimensions:

*Comprehension* (C), which includes I and TA;
Reflection on content (CR);
Reflection on processes (PR);
*Critical Reflection* (CRIT), which coincides with PREM.

The rationale for this choice is linked, first and foremost, to the specific objectives of our study, aimed at concentrating its analyses on the properly reflexive dimensions (reflection on content/process/criticism) to

bring out the aspects of tacit knowledge and implicit learning that are inherent in the explicit dimension of thought and knowledge;
explore what triggers change and perspective transformation associated with the development of genuine reflection on premises.

To this end, it proved more functional to simplify the scale by grouping the non-reflective levels (I and TA) into a single dimension, that of *Comprehension*, a type of learning characterized by the “Use of existing knowledge without attempting to appraise that knowledge, so learning remains within pre-existing meaning schemes and perspectives” (Kember et al., 2000, p. 384). This dimension also includes the affective aspects that were excluded by Kember due to the privilege given to the cognitive components of reflectivity. In our scale, comprehension encompasses the affective domain and, therefore, the dimensions of Introspection and Thoughtful action, in which understanding takes place without relating to other situations (Bloom, 1979).

Secondly, the choice was made to eliminate Kember et al.’s (1999) hybrid clustering of ‘content and process reflection’ (CPR) and to keep the two dimensions distinct, even though—as is normal—they may often be co-present in the same diaristic writing.

This is because, on the one hand, in accordance with the observations of Bell et al. (2011)^6^, it is not considered useful to apply too strictly a hierarchical view in coding the dimensions of reflexivity. It is evident, in fact, that different reflexive strategies can be combined in various ways depending on the different contexts, and it is not necessarily the case that reflection on premises is always the best choice, where the hermeneutic framework of a given reflection is considered the most functional or valid by the subject. Moreover, change and transformation—albeit on a less profound and impactful level—also occur in forms of reflexivity and non-reflexivity other than the critical one, which should always be kept in mind and valued. Nevertheless, it is also clear that, in terms of ontogeny of thought and transformative impacts, critical reflexivity is the highest attainable level. The interest in our study, in this case, was in mapping not so much the interactions between the different levels of reflexivity, but the highest level of reflexivity expressed in each diaristic writing (not necessarily considering it or having to evaluate it as the best among possible options). Therefore, in the case of the co-presence of several forms of reflexivity (or non-reflexivity), the highest level was coded.

This choice, on the other hand, also had the advantage of enabling the researchers to delineate more precisely the difference between the individual reflexive strategies, with the aim of bringing out the differences between those more oriented towards objective reframing (“reflection on what”) and those more related to subjective reframing (“reflection on how”). This was done to test the hypothesis, fundamental to the study, that it is possible to identify a preliminary threshold to the full unfolding of critical reflexivity, preceding it and identifiable precisely in the passage from content reflexivity to process reflexivity.

Table 2 summarizes the different levels of reflexivity according to Kember et al. (1999), and the categorization adopted in this paper.

### 4.4. Results of the Coding on Levels of Reflexivity

Each diaristic entry was independently coded by two coders based on the previously illustrated 4-dimensional coding grid. Cohen’s Kappa index was used to assess inter-rater reliability. The outcome showed significantly high concordance values between the two coders (K = 0.758, *p* < 0.001).

Analyses conducted on the overall sample of 62 diaristic entries showed a fairly homogeneous distribution across the first three dimensions of reflexivity taken into consideration: 20 diaristic entries showed *non-reflexive strategies* (*Comprehension*), amounting to 32.3% of the sample. Below is an example of a non-reflective strategy extracted from a diaristic entry:

“*When I finished my work, the project manager in charge of me decided to revise the logic that I had developed with effort and commitment. He wanted me to change it completely.*”

In this case we are dealing with *thoughtful action*: description, focused attention, without attribution of meaning:

“*This brought me tension, after my big effort and work, I had to rewrite everything! (…) The tension this brought me did not last long, although, in the first hours after it happened, it took away my clarity of thought and made me work less profitably.*”

Here, the diarist describes his or her own emotional feelings/states (*Introspection*) without any critical analysis.

In total, 22.6% of the narrative productions expressed *reflexivity about the content* (a total of 14 diaries), as in the following case:

“*last week my partner and I, (with whom I have been cohabiting, in her house for about 4 years) entered into a “couple crisis”, which took me quite by surprise because, although in fact, it had been preceded for about a month and a half now, by various warnings (mood swings, quarrels for trivial reasons etc.) I essentially correlated them with the period of stress she is going through, influenced among other things by hormonal changes resulting from the premenopausal.*”

In this extract, the subject starts by describing his own emotional state of surprise and the correlates of his state of mind (dimension of *Comprehension*), due to a bad argument with his partner. Immediately afterwards he deepens his analysis, making a very detailed critical examination of the ‘objective’ reasons underlying the situation that generated the surprise:

“*I specify that she has justified the period of stress she is going through and that it basically concerns the difficulty in being able to manage the very little free time she has at her disposal to the best of her ability. Free time reduced to the bone, both in terms of work commitments and, above all, those related to the management of her 10-year-old child, who, playing football, must be accompanied to training sessions three times a week, as well as to matches which, with very rare exceptions, take place on both Saturdays and Sundays. This condition not only prevents us from being able to devote ourselves to other things (travelling or going out with friends), but also from being able to easily take care of other daily tasks. This situation essentially stems from her ex-partner’s failure to alternate in what the court has established for joint custody of the child. The child’s father, for alleged work reasons, has always shirked the obligations of the judgment and especially in these last months given the lack of notice, as to when he could possibly be with his child, does not allow us to organize anything.*”

The description is analytical and detailed and reveals an aptitude for examining the validity of lived experiences. It is also evident in the narrative that the subject considers the existing situation to be substantially unalterable, since the objective conditions cannot be changed by any intervention insisting on the factual and real aspects in the strict sense. Indeed, he concludes by saying:

“*We have ascertained that in the face of this behavior, the avenues that can be pursued from a legal point of view do not guarantee positive results, because the law, net of warnings and possible sanctions, does not in fact provide for any such obligation towards the father, and a legal action would probably further undermine the current condition.*”

There is no critical analysis of the internal subjective conditions that guide the way the experience is processed, nor an in-depth examination of ‘how’ the experience itself is interpreted and lived.

*Process reflexivity* was detected in 41.9% of the cases (26 diaries), as in this diary, in which a rather stressful meeting with the company CEO is described:

“*There were four of us: me, three colleagues and our CEO. The meeting had been called mainly by the CEO, who was worried about some data that didn’t add up. […] I felt slightly less involved than my colleagues, as my role was only tangentially related to the topics discussed. My real problem was actively participating in the discussion.*”

In this first part, the subject gives the objective and real context of the situation and a description of his own state of mind (*Comprehension*). But later he deepens the analysis and frames it from a subjective and inner process point of view:

“*I often find myself not verbalizing thoughts, waiting for others to speak before I fit in, but many times I end up doing it too late. During the meeting, I realized that some of my colleagues expected me to express more of my opinion on the issues addressed. Inside, I justified this hesitation with my youthful age and less experience, as well as the lack of sufficient data to formulate a solid opinion. By the end of the meeting, I realized that I could have contributed more and avoided appearing to be a passive observer.*”

In this case, the diarist makes a critical analysis of his or her own ways of thinking, feeling or behaving, in which the focus is on the subject and his or her ways of processing the experience (and not only on the content of the experience itself). This inner gaze brings about initial awareness that enables the person to imagine a plan of action that will bring about a change with respect to similar factual situations.

“*This awareness made me reflect on the need to change my approach. This mode of participation of mine is starting to get to me, and I think it is time to put more effort into expressing my opinions. In the future, I want to prepare better, collect relevant data and think in advance about the issues to be discussed. This will help me overcome my reticence and participate more actively and productively in meetings.*”

Reaching the threshold of process reflection opens up to the person the possibility of thinking about transformations that can take place even when objective facts and situations remain unchanged. This shows how reaching that threshold initiates a transformative process that, although not yet as profound and disruptive as that determined by the full deployment of critical reflexivity, sets the person on a path of gradual and progressive self-awareness that reveals important transformative potentialities.

Only 2 diaries out of 62 achieved the highest dimension, corresponding to *critical reflexivity* (3.2% of diary production). Here is an excerpt containing an example of the use of this reflexive strategy that proceeds in steps. First, the person recounts the events that occurred:

“*While I was smart working at my computer working on software development, I made a small mistake […] it caused a momentary malfunction on the website I was working on. […] I was shocked when I realized this and immediately corrected the error and solved the problem, which lasted 2–3 min. But I was afraid that the customer had obviously noticed it; therefore, he would have phoned the company and immediately asked for technical support and a reason for the malfunction. He is one of the most important customers for my company. There would obviously follow a ‘judgement’ against me by my company for the mishap I had just caused. I really felt gnawing in my stomach and my heart jumped into my throat. Of course, I immediately proceeded without hesitation to correct the problem. The anxiety of this episode passed immediately, within a matter of minutes, as soon as I spoke to my colleague in charge of the project, she was completely calm about what had just happened. She made sure that the site was working and did not give it any weight. After all, these things can and do happen in my job. The disruption lasted very little, and the customer didn’t even notice!*”

The description of the situation is supplemented by the thoughts and emotions felt at the time (*Comprehension*) and complemented by the comparison with the attitude and opinion of another person. This intersubjective openness proves to be very important for the continuation of the person’s reflection, which, precisely because of that confrontation, deepens and analyses its own set of reactions and thoughts in more detail:

“*But it was my colleague’s calmness and naturalness in dealing with it that immediately calmed me down, the knowledge that I had not been judged negatively and that my competences had not been questioned at all. Analyzing with hindsight what happened, I am aware that I was very good at seeing the error and resolving it immediately and in the best way. I am aware that I sometimes get anxious when there is absolutely no reason to. The ‘seriousness’ of this incident was not there, it was I who gave it thinking also that this phantom seriousness might undermine the judgement of others towards me. It is not the first time that I realize it’s all my idea.*”

The person in this extract makes a twofold reflexive shift: on the one hand, she recognizes that she has a specific way of dealing with emotions and anxiety in general that influences her vision, with explicit recognition of the existence of multiple perspectives and the possibility of integrating her own model with alternative models of thought and emotion. On the other hand, precisely as a function of and thanks to this relational confrontation, she begins to ask—in a hinted and still timid way—the question of ‘why’ she has this kind of vision of problems and of herself. She does not yet give herself a full and complete answer, but she reaches the full awareness that this depends on premises concerning her own belief and value system, which are not universalizable. This marks an ongoing perspective transformation that, if developed further, could lead to even greater changes in the person’s subjective experience and in the way they conceive of themselves and the things that happen to them.

### 4.5. Discussion

In relation to the results that emerged, some aspects appear particularly relevant to be discussed and explained in more detail.

#### 4.5.1. Observations on the Distribution of the Sample in Relation to the Clusters of Reflexivity

The distribution of the sample in relation to levels of reflexivity shows a strong preponderance of diaries classified as *process reflection*, followed by the *non-reflective* diaries, then by diaries from the *content reflection* cluster, while the presence of diaries ascribable to the level of *critical reflexivity* was very small.

This last aspect certainly needs to be discussed and understood, comparing it with other similar studies in the literature. It appears from this comparison that this type of distribution is rather common, according to what is reported, for instance, by Kember et al. (2000) or Lundgren and Poell (2016) in their systematic review:

“*It can be observed that high critical reflection frequencies occur relatively rarely. This is in line with the common understanding of critical reflection and the potential for perspective transformation, i.e., people experience a major shift in perspective together with an alteration of their deeply held beliefs. This prospective transformation does not happen every day, nor is all learning intended to produce transformation as a result*”

(Lundgren & Poell, 2016)

“*As expected, the mean scores for habitual action and critical reflection are lower than those for understanding and reflection. We accept that values may not be directly comparable […] Critical reflection requires a major shift in perspective and an alteration of deeply held beliefs, which is a difficult, time-consuming and often painful process*”.

(Strike & Posner, 1985)

The results obtained in terms of diaries that can be counted as ‘critical reflexivity’ are therefore in line with the relevant literature.

Finally, this outcome could be partly related to the very approach of the diaristic task that the research team chose to favor. In fact, it was preferred to adopt a universalist approach with broad inclusion criteria to encourage participation and the use of the instrument by even those less familiar with narrative practice. The question-guide setting therefore deliberately stressed in a non-excessive manner the components of change and transformation of one’s own point of view, to allow all participants to appreciate and test the tool according to their own specific reflexive modalities.

#### 4.5.2. Aspects of Particular Importance That Emerged in the Qualitative Analysis of the Diaries

In the setting of guiding questions proposed to the participants, the researchers deliberately included a series of stimuli aimed at probing an aspect that remains insufficiently investigated in the systematic reviews on critical reflexivity, namely *social comparison.*

These are questions 8–11 on the emotions felt (see Table 1):

“*What did the people who were with you do?*”

“*What do you think the people who were with you thought at the time?”*

“*How did the people who were with you react?*”

“*What do you think the people who were with you felt in that situation?*”

These inputs responded to the aim of investigating whether and how much the subjects, in their own reflection, took thought and/or interaction with others into account, and what kind of reflexive developments might arise from these stimuli. In many of the narratives it emerged that it was precisely from confrontation with the perspectives of others that high-level reflexivity mechanisms were activated (cf. the example of critical reflexivity given in this paper).

Even when reflection is carried out in an individual form, it can be significantly affected by the perspectives and points of view of others. Social confrontation, which can facilitate or, on the contrary, hinder the attainment of new awareness in favor of perspective change, proves to be a very important element in the dynamics of the development of symbolic–reflexive capacity.

Therefore, serious consideration must be given to the fact that the unconscious mental models, to which the belief and value systems are anchored that critical reflexivity has the task of exploring, investigating, criticizing and eventually integrating and modifying, have a strongly intersubjective nature and matrix. Psychological research, both evolutionary and psychodynamic, has extensively explored this idea (Bion, 1962; Fonagy et al., 2005; Crittenden, 2013; Ogden, 2025; Civitarese, 2023)^7^, which has also been reinforced by evidence from affective neuroscience that suggests and substantiates how the socio-cultural matrix and relational dynamics with significant figures in our experience profoundly influence the creation, but also especially the updating, of unconscious mental models (Siegel, 2021; Schore, 1994, 2001, 2003, 2019; Hill, 2017). Such updating, which, as we have seen, is central to the processes of perspective transformation of critical reflexivity, requires at least two distinct mental steps:

becoming aware of our own setting of unconscious models that define the field and constraints of our ways of thinking, acting, and relating to the physical and social environment around us;
recognizing its relativity and non-absoluteness, and thus the existence of *different* perspectives and premises that may be optional.

These two operations are the engine of critical reflection and require the assumption of a certain distance from one’s own reference models to observe them from the outside and critically evaluate them. In this distancing, the contribution offered by comparison with others is, to all intents and purposes, essential, as Mezirow (1990) himself emphasizes^8^:

“*Our formative years that have often resulted in distorted views of reality. Our meaning schemes may be transformed through reflection upon anomalies. For example, a housewife goes to secretarial school in the evening and finds to her amazement that the other women do not have to rush home to cook dinner for their husbands as she does. Perspective transformations may occur through an accretion of such transformed meaning schemes. As a result of the transformation of several specific meaning schemes connected with her role as the traditional housewife, she comes to question her own identity as predicated upon previously assumed sex stereotypes.*”

(Mezirow, 1990, p. 13)

It thus emerges from these reflections how social confrontation, especially with the most affectively significant people, can encourage (or, on the contrary, hinder) the questioning of established models and the consideration of potential alternatives from a transformative perspective.

## 5. Study 2: Reflexivity and Emotional Granularity

### 5.1. Objectives and Hypotheses

The aim of this second study is to explore whether there are differences between non-reflective and reflective diaries in terms of emotional lexicon granularity. Although the construct of emotional granularity is broad and encompasses various components of emotional experiences and how they are experienced, read, and interpreted, emotional vocabulary plays a key role in determining an individual’s emotional competence. Inferring the complexity of emotional experience through lexis is not limited to counting the number of entries attributable to the emotional lexicon within a written text. While the quantity of emotional terms is an indicator of how well the diarist is able to name his or her emotional experiences, it is not a satisfactory indicator of emotional granularity. If, for instance, a diarist used many emotional terms within a diary, but these were all ascribable to the same emotional category, or if the emotional label used was always the same, in this case the multiplicity of emotional entries would correspond to poor emotional granularity.

In contrast, emotional granularity may be better represented by the quantity of different emotional terms belonging either to the same emotional category (the ability to discern between shades of emotions of the same valence/intensity) or to different emotional categories (the ability to understand that emotions are not closed, fixed and immutable categories). Therefore, the following is hypothesized:

**HP1.**  *Diaries with more complex levels of reflexivity have more different emotional terms than diaries with less complex levels of reflexivity.*

In addition to the variety of terms used in diary productions, another essential aspect is the use of emotional quantifiers (very much, a little, more, less, frequently, etc.) which, even in the presence of a repeated emotional label, provide the understanding that the same emotion can be experienced with different intensities and gradations. Consequently, we hypothesized the following:

**HP2.**  *Diaries with more complex levels of reflexivity present more emotional quantifiers than diaries with less complex levels of reflexivity.*

In the present study, we also proposed an *Emotion lexicon granularity index* (*ELGI*), determined by the ratio of the sum of different emotional words and the number of quantifiers to the total number of emotional words. We therefore formulated a third hypothesis:

**HP3.**  *The value of ELGI is higher in diaries with more complex levels of reflexivity than in diaries with less complex levels of reflexivity.*

The hypotheses were formulated from the operationalization of the emotional granularity construct, which we will illustrate in the procedure.

### 5.2. Procedure

To ensure homogeneity and comparability of the diary productions, we excluded diaries with a total word count of less than 100. Thus, we excluded 14 diaries. The remaining 48 diaries were distributed as follows: 10 non-reflective; 12 content reflective; 24 process reflective; 2 critical reflective.

In the light of the reflections set out in the theoretical part, the corpus obtained was traced to two macro-dimensions of reflexivity that group the reflective dimensions identified by Mezirow slightly differently while maintaining the relevant distinctions:

*Reflection on what*, which includes all diaristic coding of non-reflective forms and reflection on content;
*Reflection on how*, which includes all diaristic coding of forms of reflection on process and critical reflexivity.

This approach was found to be functional in recognizing reflection on process as a fundamental dividing line for the development of critical reflexivity, a preliminary step to the full unfolding of its characteristics, but foundational for reflexivity to take its first steps. In our opinion, marking a clear threshold can help identify the perspective transformation described by Mezirow. The transformation of the how, therefore, proves to be preliminary to the question of why the subject thinks and processes things in one way rather than another, and this suggests that process reflection can be regarded as the ‘ground zero’, the inaugural threshold, of reflection on premises.

The identification of this threshold made it possible to explore what kind of relationships there are between writing, reflective thinking and emotional awareness, studied based on the construct of emotional granularity (Barrett, 2017). To operationalize the construct of emotional granularity, we considered the purely linguistic aspects that may in some way represent indices of the richness and complexity of emotional experience at lexical and semantic levels within the diaristic productions, in particular

Total number of emotional terms (including feelings, affects, mood, states of mind)
Total number of different emotional terms
Number of positive vs. negative emotional terms (valence)
Number of quantifiers (intensity: a lot, a little, etc.)

Since the diary entries required the diarists to focus on critical or otherwise problematic episodes in their lives, valence was excluded from the analysis (we would probably expect a strong imbalance with a higher frequency of negative terms).

In the present study, we also proposed a summary *index of emotional lexicon granularity* (ELGI), which is determined by the ratio between the sum of different emotional words and the number of quantifiers and the total number of emotional words. ELGI is calculated as follows:

$$\mathrm{ELGI}\;=\;\frac{different\;emotional\;terms+quantifiers}{emotional\;terms}$$

To ensure comparability between the diaries, the data from each diary was weighted by the total number of words contained in that specific diary (and multiplied by 100 to avoid infinitesimal numbers).

In addition to the emotional granularity dimension, the analysis also includes the total number of terms, the total number of different terms, the total number of cognitive terms and the total number of different cognitive terms as a check on the lexical structure of the diaries and their articulation.

### 5.3. Analysis and Results

To identify the lexical indices of emotional granularity described above, two independent coders performed textual coding of the diary entries. Having verified that our data do not meet the assumptions of normality or homogeneity of variance required for parametric tests, we proceeded to use the non-parametric Mann-Whitney U Test for independent samples on all variables involved. The averages of the data distributions for each variable between non-reflective and reflective diaries are shown in Table 3.

For all variables taken into consideration, the averages in the sample of REFLECTION ON HOW diaries are higher than those of REFLECTION ON WHAT diaries. In particular, the average total number of terms in the Reflection on how diaries is higher than the average in the Reflection on what diaries. However, although present, this difference is not significant (U = 380.5; *p* = 0.051). Regarding the total number of different terms, they are significantly more present in the REFLECTION ON HOW diaries (U = 385; *p* = 0.04).

The distribution of cognitive and cognitively different terms is also different and always greater in the REFLECTION ON HOW diaries than in the REFLECTION ON WHAT diaries (respectively: U = 385.5; *p* = 0.04; U = 382; *p* = 0.047).

Coming to the data representing the complexity of emotional experiences in terms of the granularity of the emotional lexicon, the distribution of overall emotional terms is significantly more present in the REFLECTION ON HOW diaries (U = 382; *p* = 0.047).

**Figure 1:**
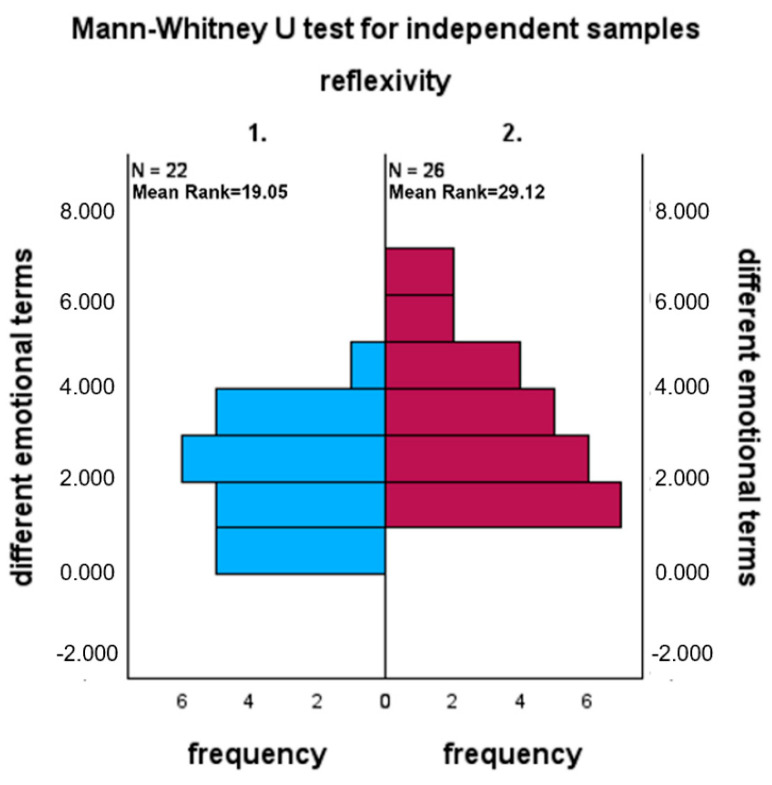
Mann–Whitney U test on differences between REFLECTION ON WHAT and REFLECTION ON HOW diaries regarding the number of different emotional terms.

About our first hypothesis, i.e., that the REFLECTION ON HOW diaries present a greater number of different emotional terms than the REFLECTION ON WHAT diaries, the test shows a different distribution of the data in the two samples, in the hypothesized direction (U = 406; *p* = 0.013). The hypothesis is therefore confirmed (Figure 1).

**Figure 2:**
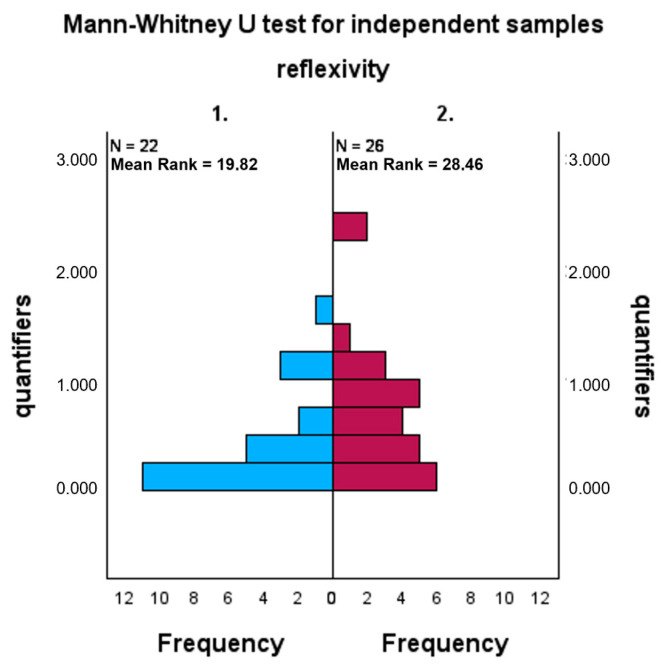
Mann–Whitney U test on differences between REFLECTION ON WHAT and REFLECTION ON HOW diaries regarding the number of emotional quantifiers.

The second hypothesis is also confirmed (Figure 2): the REFLECTION ON HOW diaries have significantly more emotional quantifiers than the REFLECTION ON WHAT diaries (U = 389; *p* = 0.031).

**Figure 3:**
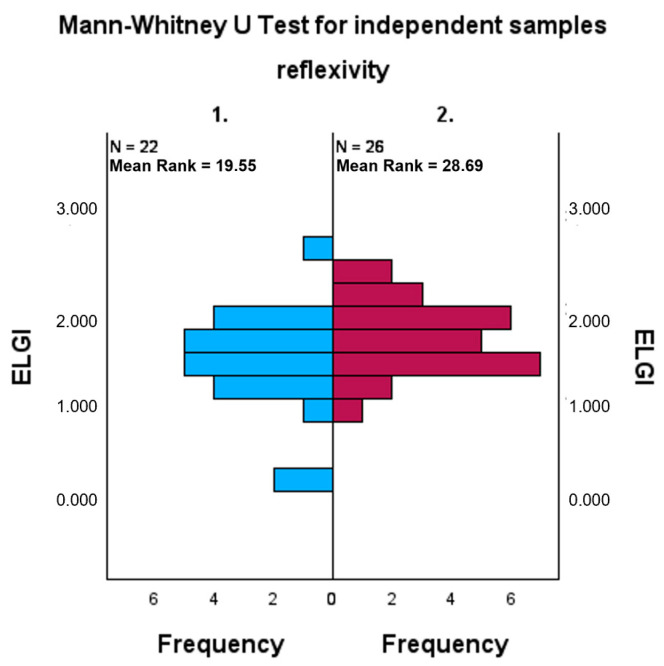
Mann–Whitney U test on differences between REFLECTION ON WHAT and REFLECTION ON HOW diaries regarding ELGI.

Finally, with reference to the third hypothesis, the ELGI synthesis index is also significantly higher in the REFLECTION ON HOW diaries than in the REFLECTION ON WHAT diaries (U = 395; *p* = 0.024). Consequently, the third hypothesis is also confirmed (Figure 3).

## 6. General Discussion and Conclusions

In the course of this paper, the theoretical and experimental analyses carried out to explore the relationship between emotions and reflexivity were reported, with particular reference to the construct of critical reflexivity, elaborated by Mezirow (1991) and operationalized by Kember et al. (2000), and to the construct of emotional granularity, proposed by Barrett (2017).

The central question that was discussed concerned the exploration of what the basic requirements/skills are on which the development of critical reflexivity is built over time, with particular attention to the role played by emotional competences in such a path of acquisition, in order to fill the gap that has emerged in the literature on this aspect, which is still little explored by researchers in the field.

This line of research was specified with the aid of an exploratory experimental investigation, guided by the following questions:

Is it possible to hypothesize the existence of a threshold preliminary to the full unfolding of critical reflexivity, which is prior to it and linked to the development of process reflexivity?
Is it possible to argue that crossing this threshold can be facilitated by the acquisition of a good level of emotional competence?

In relation to (b), the results of the exploratory study confirm the three sub-hypotheses put forward in relation to the reciprocal nurturing relationship between reflexive skills and emotional competence. The two groups analyzed (“Reflection on what” and “Reflection on how”) differ significantly with respect to all three of the variables analyzed, linked to emotional granularity, thus proving to be more competent in recognizing and expressing the emotions felt. This greater richness and ability to articulate the emotional spectrum in more specific, differentiated and contextualized forms thus seems to confirm the idea that this competence is a supporting element in the development of more complex and elevated modes of critical reflexivity.

These results—it is important to note—are primarily ascribable to the process reflexivity component, which weighs over 90% in the “Reflection on How” group. This fact brings with it two types of considerations.

The first is related to the obvious limitation represented by the fact that we do not have a large number of diaries at the higher end of reflexivity, which significantly reduces the possibility of comparing diaries coded as “process reflective” with “critical reflective” diaries in the “reflection on how” group. It would be interesting, in the future, to carry out studies focusing solely on this grouping and having as inclusion criteria, at the outset, an appropriate balance of the process and critical reflexivity components. In these studies, an increasing level of complexity in terms of instructions and guiding questions could be applied in order to bring out more clearly the specific modes of reflexivity of the sub-groups from a comparative perspective, in order to verify, among other things, whether the low distributions recorded in the highest reflexivity clusters are also partly related to tasks that are too low in terms of analyzing oneself and one’s life context.

The second emphasizes, on the other hand, how precisely the specific weight of process reflexivity with respect to the results obtained by the ‘How’ group—with the confirmation of all three hypotheses linked to the emotional granularity of the sample observed—reinforces the idea that the development of solid emotional competences is an indispensable element for the development of process reflexivity, *even before critical* reflexivity.

This means, on one hand, that the path of reflexivity tends to proceed hand in hand with that of emotional self-awareness and one’s own functioning mechanisms. On the other hand, the first idea from which the paper moves (a), that is, the possibility of identifying in process reflection a fundamental threshold of access to critical reflexivity as its condition of possibility and basic requirement, is plausible and merits further investigation in order to arrive at its verification.

With respect to the future developments of this line of research, it is pointed out that a further element that could enrich the analysis of the emotional and affective components is represented by the combination of the diaristic practice with a setting that also includes interviews, in which the non-verbal and para-verbal components of interaction can also be examined. In the study carried out, these were not included, which certainly represented a limitation from the point of view of the information that could be gathered.

The adoption of more interactive ways of assessing reflexivity should also be evaluated, which can be captured, for example, through experiential assessment formats that, with the aid of combined qualitative observation techniques in presence and video-recording, can also make it possible to assess the relational dynamics at play, in addition to the emotional and non-verbal components. The “assessment” modality would also make it possible to construct real critical situations/case studies to test the different modes of reflexivity according to a laboratory approach, in which it would be possible to isolate the different variables and make the behavior observed in the sample more comparable and controllable.

Although the results presented in this paper show encouraging outcomes regarding the connections between reflective thinking and emotional competence, certain limitations make further investigation necessary. First, the number of participants is extremely small, and the data obtained from the analysis of the diaries can be interpreted as trends that require stronger statistical confirmation in future research. This aspect makes it necessary to consider this contribution as an exploratory study, considerably limiting the generalizability of the results.

Another aspect to consider is related to the sample selection phase. No pre-assessment was conducted to verify the participants’ levels of reflectivity, and it is possible that subjects with a predisposition to reflectivity, or simply those familiar with diary techniques, were more likely to participate in the study. The effect of this bias was partially mitigated during the information phase by providing all subjects with clear and simple information on how to complete the diaries. Guidelines to guide participants in the reflective writing technique were provided to help diarists exercise critical and reflective thinking.

In conclusion, in the light of what has emerged from this preliminary study, it is plausible to argue that transformative learning theory, in order to advance the understanding of the phenomena and dynamics underlying critical reflexivity, would benefit from the in-depth study of the following lines of research:

The exploration of the emotional and affective components of the non-reflective and reflexive processes that support the development of critical reflexivity, through theoretical, methodological and experimental settings capable of assessing these aspects, including formats dedicated to a focused in-depth understanding of the dynamics linked to the individual dimensions of reflexivity (in particular, the transition from content reflection to process reflection);
The analysis of the socio-cultural components and dynamics that influence the development of critical reflexivity, through the adoption of analysis formats (theoretical and experimental) that overcome the constraints and limits of the individual approach (in which the subject reflects alone or without the impact of interactions with others, real or internalized).

Indeed, the analyses conducted thus far, however preliminary and exploratory, indicate that these directions can provide new impetus for studies in this field and stimulate interdisciplinary confrontation between different domains of knowledge and research on a subject that has significant applicative potential. In the case of the empirical study cited, for example, the applicability of narrative reflexive technologies to the field of psychological well-being and stress management in professional contexts within private and public organizations was explored. It is not implausible to think of further potential future developments of this approach that would go as far as to test itself on larger populations, with mixed reflective formats (diaries, interviews, critical incidents) with a view to research, but also to training and the promotion of organizational empowerment (Tossici, 2020).

## Figures and Tables

**Table 1:** Guidelines on the reflective writing technique.

Have you recently felt tense about something or faced particularly difficult situations? Describe your experience in narrative form, including ***1.*** ***Description of the situation***—describe the event that caused your tension/difficulty ***2.*** ***The emotions you felt***—when describing them, you can use the following descriptors: “When did this episode occur?” “Where did this episode occur?” “Who was with you?” “What happened?” “What did you do?” “What did you think at that moment?” “How did you react?” Try to describe the physical sensations you experienced during that emotion; “What did the people who were with you do?” “What do you think the people who were with you were thinking at that moment?” “How did the people who were with you react?” “What do you think the people who were with you felt in that situation?” ***3.*** ***Solutions implemented*** “What did you do to manage the situation?” “What did you do to manage your emotions?” ***4.*** ***Re-evaluation of events***—now that you have described what happened, try to reflect on these aspects: “Are there any positive aspects to the situation you experienced? If so, what are they?” “Did you achieve any goals in that situation? What were they?” “Did the anxiety or tension you felt hinder you or help you achieve these goals? How?” “Did the emotions you felt hinder you or help you achieve these goals? How?” **The use of all descriptors is not mandatory** but consider them useful ideas for developing your narrative. It is important that you write spontaneously whatever comes to mind, without worrying about syntax and grammar.

**Table 2:** Reflexivity levels according to Kember et al. (1999) and our proposal.

Author	Reflexivity Levels	Reflexivity Sub-Levels	Description
Kember	Non-reflective action	*Habitual action (HA)*	Habitual action is that which has been learnt before and through frequent use becomes an activity which is performed automatically or with little conscious thought (Kember et al., 1999).
		*Introspection (I)*	Introspection lies in the affective domain. It refers to feelings or thoughts about ourselves, or feelings towards others. Introspection remains at the level of recognition or awareness of these feelings (Kember et al., 1999).
		*Thoughtful action (TA)*	Thoughtful action makes use of existing knowledge, without attempting to appraise that knowledge, so learning remains within pre-existing meaning schemes and perspectives. Thoughtful action can be described as a cognitive process of knowledge, comprehension, application, analysis and synthesis (Kember et al., 1999).
	Reflective action	*Content reflection (CR)*	Content reflection is concerned with *what*, and reflection on what we perceive, think, feel or act upon (Kember et al., 1999).
		*Process reflection (PR)*	Process reflection is concerned with *how*, that is our method or manner in which we think: examination of how one performs the functions of perceiving, thinking, feeling, or acting and an assessment of efficacy in performing them (Kember et al., 1999).
		*Content and process reflection (CPR)*	Combined category including examples of reflections where content and process are inextricably linked (Kember et al., 1999).
		*Premise reflection (PREM)*	Premise reflection is concerned with a significant change in perspective. It involves our becoming aware of why we perceive, think, feel or act as we do (Kember et al., 1999).
Our proposal	Non-reflective action	*Habitual action (HA)*	Excluded because it is not relevant to diary entries.
		*Comprehension (C)*	“Use of existing knowledge without attempting to appraise that knowledge, so learning remains within pre-existing meaning schemes and perspectives” (Kember et al., 2000, p. 384). It includes the affective domain and therefore the dimensions of Introspection and Thoughtful action, in which understanding takes place without relating to other situations (Bloom, 1979).
	Reflective action	*Content reflection (CR)*	Content reflection is concerned with *what*. ‘Reflection on what we perceive, think, feel or act upon’. (Kember et al., 1999; citing Mezirow, 1991, p. 107)
		*Process reflection (PR)*	Process reflection is concerned with *how*, that is our method or manner in which we think. ‘Examination of how one performs the functions of perceiving, thinking, feeling, or acting and an assessment of efficacy in performing them’ (Kember et al., 1999; citing Mezirow, 1991, pp. 107–108).
		*Critical reflection (CRIT)*	Critical reflection is concerned with a significant change in perspective. It ‘involves us becoming aware of why we perceive, think, feel or act as we do’ (Kember et al., 1999; citing Mezirow, 1991, p. 108). It coincides with the premise reflection of the classification by Mezirow (1991) and Kember et al. (1999).

**Table 3:** Data distributions for each variable between non-reflective and reflective diaries.

Reflexivity		n Terms	n Diff. Terms	Cognitive Terms	Diff. Cognitive Terms	Emotion Terms	Diff. Emotion Terms	Quantifiers	ELGI
1 non-reflective	Mean	227.82	142.59	2.77323	3.61764	1.73550	2.10255	0.38727	1.33786
	N	22	22	22	22	22	22	22	22
	Std. Deviation	119.963	55.648	1.547319	2.266635	0.993984	1.229696	0.475634	0.577363
2 reflective	Mean	300.35	181.42	3.55281	4.46808	2.55492	3.29369	0.72900	1.65773
	N	26	26	26	26	26	26	26	26
	Std. Deviation	157.109	74.423	1.756623	2.055153	1.220317	1.497612	0.611180	0.354695
Total	Mean	267.10	163.63	3.19550	4.07829	2.17935	2.74775	0.57238	1.51113
	N	48	48	48	48	48	48	48	48
	Std. Deviation	144.545	68.645	1.692685	2.173830	1.184823	1.492776	0.573917	0.491735

## Data Availability

The research data is confidential and cannot be made publicly available. The participants’ anonymity has been guaranteed, and the protocols contain sensitive data that could lead to the identification of participants if made public. This material will be available to interested researchers upon request and upon signing informed consent.

## Notes

1 *Tacit knowledge* refers to the set of skills possessed by the individual, often unconsciously or unreflectively, which are drawn from non-formalized learning and which guide the individual’s personal and professional behavior and practices (Polanyi, 1996). This implicit bearing influences both the technical practices, i.e., the set of skills that identify the ‘know-how’ of habitual activities, and the cognitive components of our experience, i.e., the set of schemas, mental models, beliefs and perceptions, relating to reality and the world around us, that are so consolidated as to have become axiomatic, representing the tip of the iceberg of the overall body of knowledge (Arzuffi et al., 2011); a description in line with Mezirow’s notion of the ‘premises’ of critical reflexivity.

2 Siegel (2021) defines them as engrammas that play a fundamental role in our learning processes: derived from the past, they influence our perceptual experiences of the present and help us to intuit the future—and to act accordingly. The ability to ‘anticipate’, at least in part, what the future holds for us can be regarded as an essential component of implicit memory.

3 LTP is a particular physiological process that occurs with an increase in signal transmission between two neurons and is characterized by a series of molecular changes in the function and microstructure of one or more synaptic junctions when the two neurons are stimulated in a long-term manner.

4 The pathway of rediscovering and reworking these schemata is very akin to the objectives of the psychotherapeutic pathway that aims to lead the patient to re-evaluate his or her orientation to perceiving, knowing, believing, feeling and acting, in order to bring out the psycho-cultural assumptions that inform his or her ways of viewing and processing reality (Van Woerkom, 2010).

5 Emotional granularity is also known as “emotional complexity,” “emotional differentiation,” or “emodiversity”.

6 Bell et al. (2011) in their critical review discuss the assumption that critical reflexivity is always the best option, as it is of a higher degree: “we question whether a densely reflective journal that focuses primarily on content reflection is of less quality than a journal that contains one premise (critical) reflection. Grading according to highest level of reflection doesn’t represent the broad spectrum of reflection that may be encompassed in the student’s writing. We see the categorization scheme of Kember et al. (1999) as an integrated whole, rather than as ‘levels’ of reflection. Kember et al. (2000) show that the categories are indeed inter-correlated, but that finding does not seem to have been carried across to the grading in Kember et al. (2008). Reflection isn’t only about transformation. Why should a student have to change as a person to get a high grade? A student could learn a great deal in a course but their perspective might already be aligned with what is being taught, and their views might be validated rather than changed” (Bell et al., 2011, p. 809).

7 The psychological and neuroscientific literature on intersubjectivity is vast and cannot be summarized in a few lines. Among the most interesting and potentially relevant hypotheses for an understanding of the socio-cultural foundations of reflexive processes, we would like to point out the one put forward by authors who move in the wake of second-generation attachment theory, such as Crittenden (2013) and Fonagy et al. (2005), according to whom the true adaptive purpose of primary relationships may lie precisely in the child’s learning of a system for processing information, as well as for regulating affects: “The genesis of the representational system that forms an active filter between genotype and phenotype is intrinsically linked to the quality of early object relations. Perhaps more than shaping the quality of subsequent relationships (a fact for which evidence is lacking), the primary relational environment serves to equip the individual with an *information processing system.* The creation of this information representation system is, one might say, the most important evolutionary function of caregiver attachment” (Fonagy et al., 2005, p. 76). These learnings, which take place at a very early age, endow the individual with a “thought apparatus” that forms the functional basis of all subsequent processing of experience and which, we hypothesize here, may represent the hermeneutic framework of the higher-order reflexive processes discussed at length in this paper.

8 Mezirow’s example expresses the concept very well here. If, in fact, everyone in our social environment shared a single model setting, it would be much more difficult to make the comparison that allows one to evaluate different options with respect to one’s own established models. Diversity, in this, proves to be a key factor for change and evolution of one’s personal perspectives, as research and practices related to Diversity Management have gradually highlighted over time.
